# A systematic analysis of immune genes and overall survival in cancer patients

**DOI:** 10.1186/s12885-019-6414-6

**Published:** 2019-12-16

**Authors:** Qian Wang, Pan Li, Weidong Wu

**Affiliations:** Center for Public Health Informatics, School of Public Health, Xinxiang Medical University, Xinxiang, Henan People’s Republic of China

**Keywords:** Overall survival, The immune system, PIGs, T cell receptor signaling pathway

## Abstract

**Background:**

Overall survival (OS) is a key endpoint measure in the management of patients with cancer. Immunotherapy has become a dominant strategy in cancer therapy. To investigate the relationship between OS and the immune system, we assessed the role of immune genes in OS in 8648 patients across 22 cancer types.

**Methods:**

Gene expression data and clinical information were collected from The Cancer Genome Atlas (TCGA) and cBioPortal. Survival analysis was performed with a Cox proportional hazards regression model.

**Results:**

(1) The number of prognostic genes, prognostic immune genes (PIGs) and the hazard ratio (HR) of PIGs in different cancer types all varied greatly; (2) KEGG pathway enrichment analyses indicated that the prognostic genes of 6 cancer types were significantly enriched in multiple (≥5) immune system-related pathways. Of the PIGs in these 6 cancer types, we screened 48 common PIGs in at least 5 cancer types. Eleven out of the 48 PIGs were found to participate in the T cell receptor (TCR) signaling pathway according to the STRING database. Among these genes, ZAP70, CD3E, CD3G, CD3D, and CD247 were part of the TCR ‘signal-triggering module’; (3) High expression of the PIGs involved in the TCR signaling pathway was associated with improved OS in 5 cancer types (breast invasive carcinoma (BRCA), cervical squamous cell carcinoma and endocervical adenocarcinoma (CESC), head and neck squamous cell carcinoma (HNSC), lung adenocarcinoma (LUAD), and sarcoma (SARC)), but was associated with decreased OS in brain lower-grade glioma (LGG).

**Conclusions:**

The TCR signaling pathway played a distinct role in the OS of these 6 cancer types.

## Background

Cancer is the leading cause of death worldwide [1–3]. Over the past decade, the incidence of cancer has increased rapidly with an aging population, and the increasing prevalence of established risk factors such as smoking, overweight, and physical inactivity [2]. Although some progress has been made for cancer therapeutics, patients with cancer continue to experience significant morbidity and mortality [4].

Advances in the understanding of the molecular mechanisms of interaction between the tumor and immune system have provided new approaches to cancer treatment [5–7]. The most effective way to activate therapeutic antitumor immunity is the blockade of immune checkpoints [6, 8]. Immune checkpoints are regulators that play crucial roles in maintaining self-tolerance, which prevents the immune system from attacking cells indiscriminately [8]. Previous studies have demonstrated that tumor cells escape from an immune attack by activating certain immune checkpoints, such as cytotoxic T lymphocyte-associated antigen 4 (CTLA-4) and the programmed cell death protein 1 (PD-1), to resist T cell-mediated antitumor immunity [8, 9]. Since these immune checkpoints are initiated by ligand-receptor interactions that are easily blocked by antibodies or modulated by recombinant forms of ligands or receptors, they are attractive drug targets for cancer therapeutics [8].

OS is defined as the time from entry into a clinical trial until death from any cause [10]. The OS is the gold standard for evaluating the outcome of drug treatment [11, 12], surgery [13], immunotherapy [14] and biologic or other interventions in oncology clinical trials [15]. A large body of evidence has demonstrated that immune checkpoint therapy is correlated with improved patient OS in several cancer types [16]. For example, Hodi et al. demonstrated that ipilimumab improved OS in patients with previously treated metastatic melanoma [17]. Gettinger et al. found that nivolumab monotherapy produced durable responses and encouraging survival rates in patients with non-small-cell lung cancer [18]. In addition, pembrolizumab was associated with a significantly longer OS for platinum-refractory advanced urothelial carcinoma than standard therapy [19]. To date, there have been 6 immune checkpoint inhibitor antibodies (ipilimumab [17], nivolumab [18], pembrolizumab [19], avelumab [20], atezolizumab [21], and durvalumab [22]) against CTLA4 or PD-1 approved by the U.S. Food and Drug Administration (FDA) for the treatment of a few cancer types. Cancer immunotherapy has already become a component of standard cancer treatments, which include surgery, radiation, chemotherapy, and targeted therapy [23].

However, until now, the relationship between the immune system and OS across a range of cancer types has remained incompletely understood, which has made it difficult for investigators to choose the appropriate combinations of immunotherapies for each particular cancer. The availability of high-throughput datasets and clinical information over large, well-characterized patient sample cohorts of multiple cancer types from TCGA [24] provides an unprecedented opportunity to explore the relationship between the immune system and OS. Therefore, in this study, we collected gene expression datasets from TCGA [24] and clinical data from cBioPortal [25] of 22 cancer types to explore the relationship between OS and immune genes. Additionally, we identified the immune-related pathways enriched in the prognostic genes and obtained key PIGs.

## Methods

### Data collection

Gene expression datasets of all cancer types (with the substring “Level_3_RSEM_genes_normalized” in file names) were collected and downloaded from the Broad Institute’s Genome Data Analysis Center (GDAC) (http://gdac.broadinstitute.org/). These datasets are all preprocessed RNA sequence from the TCGA database and standardized by the RSEM algorithm. Clinical data were collected from the cBioPortal for Cancer Genomics (http://www.cbioportal.org/) [25]. The data selection criteria were as follows: (1) the number of samples in each dataset must be ≥100; (2) all datasets must contain clinical data; (3) and OS months and OS status clinical data in all datasets must be available. Based on the above criteria, 22 datasets (bladder urothelial carcinoma (BLCA), BRCA, CESC, colon adenocarcinoma (COAD), esophageal carcinoma (ESCA), glioblastoma multiforme (GBM), HNSC, kidney renal clear cell carcinoma (KIRC), kidney renal papillary cell carcinoma (KIRP), LGG, liver hepatocellular carcinoma (LIHC), LUAD, LUSC, ovarian serous cystadenocarcinoma (OV), pancreatic adenocarcinoma (PAAD), prostate adenocarcinoma (PRAD), sarcoma (SARC), skin cutaneous melanoma (SKCM), stomach adenocarcinoma (STAD), thyroid carcinoma (THCA), thymoma (THYM) and uterine corpus endometrial carcinoma (UCEC)) were chosen for analysis. The details of the datasets are shown in Table 1.

**Table 1:** Results of survival analysis in 22 kinds of cancer

Cancer name	Samples^a^	PGs^b^ (FDR < 0.05)	PIGs^c^ (HR > 1)	PIGs^d^ (HR < 1)	Percent^e^ (PIGs/PGs)
Bladder Urothelial Carcinoma (BLCA)	408	3423	156	148	8.88%
Breast Invasive Carcinoma (BRCA)	1095	1973	42	240	14.29%
Cervical Squamous Cell Carcinoma and Endocervical Adenocarcinoma (CESC)	304	2268	115	198	13.80%
Colon Adenocarcinoma (COAD)	285	1725	113	36	8.64%
Esophageal Carcinoma (ESCA)	184	10	0	0	0.00%
Glioblastoma Multiforme (GBM)	169	1336	119	30	11.15%
Head and Neck Squamous Cell Carcinoma (HNSC)	520	3420	143	254	11.61%
Kidney Renal Clear Cell Carcinoma (KIRC)	533	9958	616	332	9.52%
Kidney Renal Papillary Cell Carcinoma (KIRP)	290	4229	286	109	9.34%
Brain Lower Grade Glioma (LGG)	534	9359	716	281	10.65%
Liver Hepatocellular Carcinoma (LIHC)	371	3697	166	203	9.98%
Lung Adenocarcinoma (LUAD)	517	3865	138	310	11.59%
Lung Squamous Cell Carcinoma (LUSC)	501	1017	93	17	10.82%
Ovarian Serous Cystadenocarcinoma (OV)	309	1260	68	43	8.81%
Pancreatic Adenocarcinoma (PAAD)	178	4461	228	145	8.36%
Prostate Adenocarcinoma (PRAD)	497	2	0	0	0.00%
Sarcoma (SARC)	263	2750	74	284	13.02%
Skin Cutaneous Melanoma (SKCM)	472	8	0	0	0.00%
Stomach Adenocarcinoma (STAD)	415	2641	236	39	10.41%
Thymoma (THYM)	120	1	0	0	0.00%
Thyroid Carcinoma (THCA)	505	1	0	0	0.00%
Uterine Corpus Endometrial Carcinoma (UCEC)	176	17	1	0	5.88%

^a^The number of samples

^b^The number of all prognostic genes (PGs) with FDR (adjusted *p*-value) < 0.05

^c^The number of risk PIGs (HR > 1) with FDR (adjusted *p*-value) < 0.05

^d^The number of protective PIGs (HR < 1) with FDR (adjusted *p*-value) < 0.05

^e^The ratio of all PIGs to all PGs with FDR (adjusted p-value) < 0.05

The Immunology Database and Analysis Portal System (ImmPort) (https://immport.niaid.nih.gov) is a critical repository for immunology-related clinical and molecular data [26]. InnateDB (http://www.innatedb.ca/) is a publicly available database of the genes, proteins, experimentally verified interactions and signaling pathways involved in the innate immune response to microbial infection in humans, mice, and bovines [27]. Lists of human immune genes were collected and downloaded from these two databases. After merging and eliminating duplication, 2514 immune genes were identified.

### Identification of prognostic genes and PIGs

To identify the prognostic genes of each cancer type, first, patients with both gene expression data and clinical information were selected. Second, according to the gene expression levels, all samples of each gene of each cancer type were divided into three equal tertiles: samples with low gene expression level; samples with intermediate gene expression level; and samples with high gene expression level. Then, the high expression levels group and the low expression levels group were screened for survival analysis with a Cox proportional hazards regression model. HR was the hazard rate ratio of OS between a group of patients with high gene expression levels and a control group with low gene expression levels. HR > 1 indicated that high-level expression of a gene correlated with a decreased OS, and HR < 1 indicated that high-level expression of a gene correlated with prolonged OS. For the correction of multiple-hypothesis testing, the p.adjust function (R, 2013) with the false discovery rate (FDR) method was used to identify prognostic genes with a false discovery rate (FDR) (adjusted *p*-value) <0.05. PIGs for each cancer were generated by the intersection of prognostic genes and genes in the human immune gene list.

### The expression of PIGs

To explore the expression of PIGs in cancer tissues compared to normal tissues, 14 of 22 cancer types (BLCA, BRCA, COAD, ESCA, HNSC, KIRC, KIRP, LIHC, LUAD, LUSC, PRAD, STAD, THCA, and UCEC) with >10 control samples were selected and analyzed. Differentially expressed gene analyses between the case group and the control group were conducted using the empirical Bayes algorithm (the function “eBayes” in R) with an FDR for *p*-values adjustment. Differentially expressed genes (DEGs) (upregulated or downregulated) had an FDR (adjusted *p*-value) < 0.05 and an absolute FC (fold change) ≥ 1.5. By comparing DEGs with PIGs in every cancer type, the differentially expressed PIGs were obtained, as shown in Additional file 4: Table S3.

### Gene set enrichment analysis

The “phyper” function (R, 2013) based on hypergeometric distribution method was used to conduct the enrichment analysis of prognostic genes among 22 cancer types. R code was as flowing:

$$ \mathrm{P}\left(\mathrm{X}\ge \mathrm{k}\right)=1-\mathrm{phyper}\ \left(\mathrm{k}-1,\mathrm{m},\mathrm{N}-\mathrm{m},\mathrm{n}\right). $$

Where N is the number of all genes in every dataset of the 22 cancer types, n represents the number of prognostic genes in every dataset of the 22 cancer types, m is the number of all genes in the enriched KEGG pathway, k is the number of prognostic genes in the KEGG pathway. The p.adjust function (R, 2013) with the false discovery rate (FDR) method was used for multiple comparison. Significantly enriched biological pathways with an FDR (corrected *p*-value) ≤ 0.05 were selected. The enrichment percentage in each pathway was calculated as the number of prognostic genes divided by the number of all genes.

### Protein functional annotation of key PIGs

The STRING database provides a critical assessment and integration of protein-protein interactions, including direct (physical) and indirect (functional) associations, on a global scale [28]. In this study, the STRING database was used to provide a critical assessment and integration of protein-protein interactions encoded by the 48 key prognostic genes identified in 6 cancer types.

## Results

### Overview of prognostic genes and PIGs in 22 cancer types

The resulting prognostic genes and PIGs are shown in Table 1. The number of prognostic genes varied greatly with the cancer type, ranging from 1 to 10,000. KIRC and LGG had the highest numbers of prognostic genes (FDR < 0.05), which were 9958 and 9359, respectively. In contrast, THYM, THCA, PRAD, and SKCM had the lowest numbers of prognostic genes (FDR < 0.05), which were all less than 10. The number of PIGs in the 22 cancer types was consistent with that of prognostic genes. KIRC and LGG had the highest numbers of PIGs, and there were no PIGs in THYM, THCA, PRAD, ESCA, and SKCM. The ratio of PIGs to all prognostic genes in every cancer was calculated. Among the 22 cancer types, the proportion of PIGs in 9 cancer types (BRCA, CESC, SARC, LUAD, HNSC, GBM, LUSC, LGG, and STAD) was higher than 10%. Of these 9 cancer types, the proportion of PIGs in BRCA was highest, 14.29%.

### HR of PIGs varied greatly with cancer type

Previous studies have demonstrated that the immune system acts as a significant barrier to tumor formation and progression in humans, except for some forms of nonvirus-induced cancer [29]. However, in this study, PIGs were not fully protective factors in cancer. The HR of the PIGs in 22 cancer types varied greatly, as shown in Table 1. In 5 cancer types (BRCA, CESC, HNSC, LUAD, and SARC), the number of protective PIGs (HR < 1) was apparently higher than that of risk PIGs (HR > 1), and the proportion of protective PIGs (HR < 1) in total PIGs was greater than 60%. In 2 cancer types (BLCA and LIHC), there was no significant difference between the number of protective PIGs (HR < 1) and risk PIGs (HR > 1). In 9 cancer types (COAD, GBM, KIRC, KIRP, LGG, LUSC, OV, PAAD, and STAD), the number of protective PIGs (HR < 1) was significantly lower than that of risk PIGs (HR > 1), and the ratio of risk PIGs (HR > 1) to total PIGs was over 60%.

### Six cancer types were significantly enriched in immune system-related pathways

**Fig. 1:**
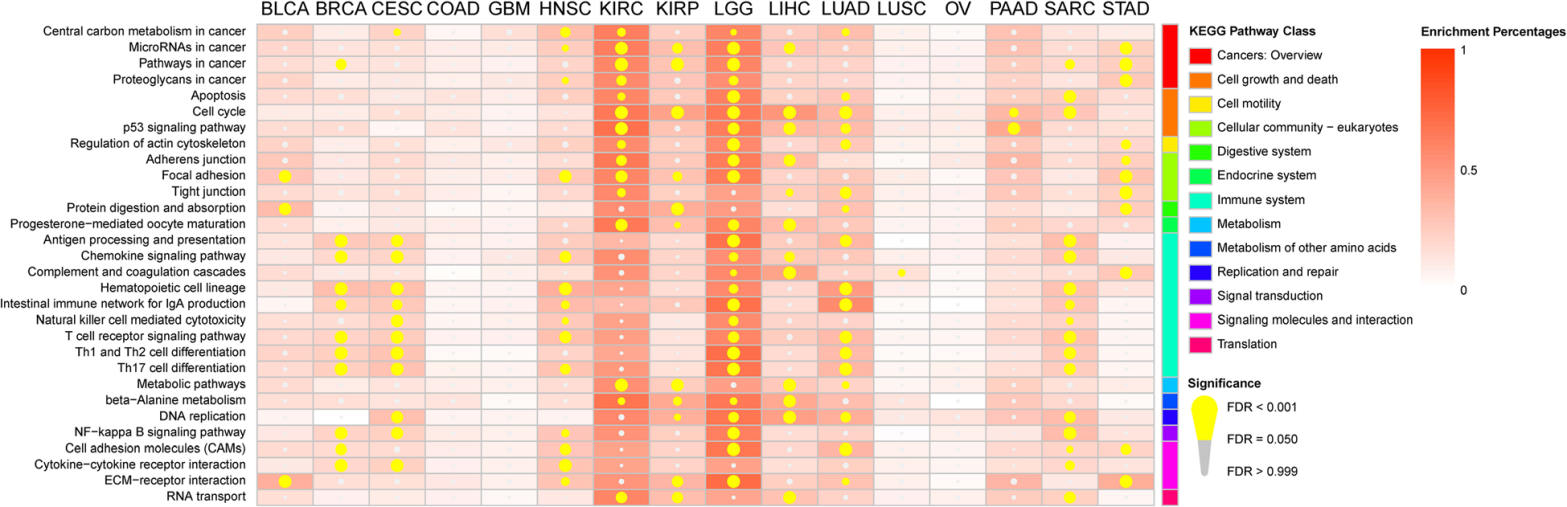
Thirty KEGG pathways were enriched in the prognostic genes with an FDR (corrected *p*-value) < 0.05 of ≥4 of 22 cancer types. Five or more immune system-related KEGG pathways (turquoise bar) were significantly enriched in the prognostic genes of 6 cancer types (BRCA, CESC, HNSC, LGG, LUAD, and SARC). Level of redness indicates the enrichment percentage of prognostic genes in each KEGG pathway. The enrichment percentage obtains from the number of prognostic genes divided by the number of all genes of each KEGG pathway

The results of gene set enrichment analysis demonstrated that the prognostic genes of 22 cancer types were enriched in 173 KEGG pathway terms (FDR < 0.05). The details are presented in Additional file 2: Table S1. The 30 KEGG pathways terms (Fig. 1) shared by ≥4 cancer types could be mainly divided into six major categories: (1) cancers (pathways in cancer, proteoglycans in cancer, microRNAs in cancer and central carbon metabolism in cancer); (2) immune system (chemokine signaling pathway, complement and coagulation cascades, antigen processing and presentation, hematopoietic cell lineage, natural killer cell-mediated cytotoxicity, Th1 and Th2 cell differentiation, Th17 cell differentiation, TCR signaling pathway and intestinal immune network for IgA production); (3) cell growth and death (cell cycle, p53 signaling pathway, and apoptosis); (4) cell communication (focal adhesion, tight junction and adherens junction); (5) signaling molecules and interaction (cytokine-cytokine receptor interaction, ECM-receptor interaction and cell adhesion molecules); (6) and other categories including cell motility (regulation of actin cytoskeleton), digestive system (protein digestion and absorption), endocrine system (progesterone-mediated oocyte maturation), metabolism of other amino acids (beta-alanine metabolism), signal transduction (NF-kappa B signaling pathway), and translation (RNA transport).

The prognostic genes of six (BRCA, CESC, HNSC, LUAD, SARC, and LGG) out of the 22 cancer types were significantly enriched in ≥5 immune system-related pathways (chemokine signaling pathway, complement and coagulation cascades, antigen processing and presentation, hematopoietic cell lineage, natural killer cell-mediated cytotoxicity, Th1 and Th2 cell differentiation, Th17 cell differentiation, TCR signaling pathway and intestinal immune network for IgA production), which are presented in Fig. 1. This result suggested that there was a strong correlation between OS and immune genes in these 6 cancer types.

### The diversity of the HRs of the 48 PIGs shared by the 6 cancer types

Recent evidence highlights that tumor-infiltrating activated T cells are associated with a good prognosis in head and neck squamous cell carcinoma [30], breast cancer [31], and non-small-cell lung cancer [32]. In this study, the PIGs of 6 cancer types (BRCA, CESC, HNSC, LUAD, SARC, and LGG) were all over 10%, and prognostic genes were also significantly enriched in ≥5 immune system-related pathways. Therefore, the 6 cancer types were subjected to further analyses. By intersecting the PIGs of the 6 cancer types, 48 mutual PIGs were identified in at least 5 cancer types. In these 5 cancer types (BRCA, CESC, HNSC, LUAD, and SARC), the PIGs shared by the 6 cancer types were all protective PIGs (HR < 1). However, most of the PIGs shared by the 6 cancer types were risk PIGs (HR > 1) in LGG. For instance, the number of common PIGs in LGG was 42, 36 of which were risk PIGs. The details are presented in Additional file 3: Table S2.

### TCR signaling pathway plays a distinct role in the 6 cancer types

**Fig. 2:**
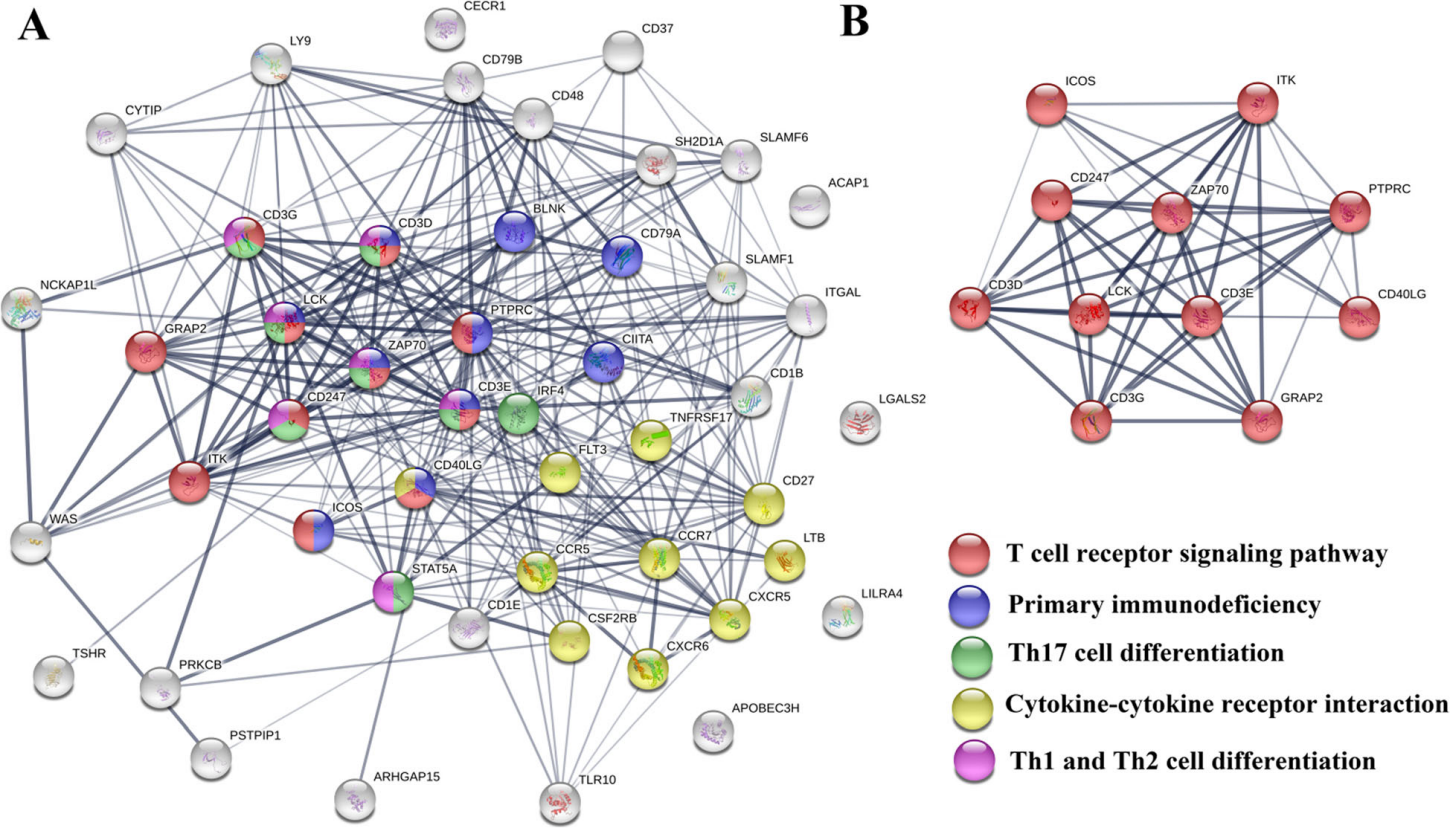
The results of protein-protein interactions were identified with STRING database. **a** Interactions of the proteins encoded by the 48 PIGs, which were shared ≥5 of 6 cancer types (BRCA, CESC, HNSC, LUAD, SARC, and LGG). **b** Eleven genes participated in the TCR signaling pathway

**Fig. 3:**
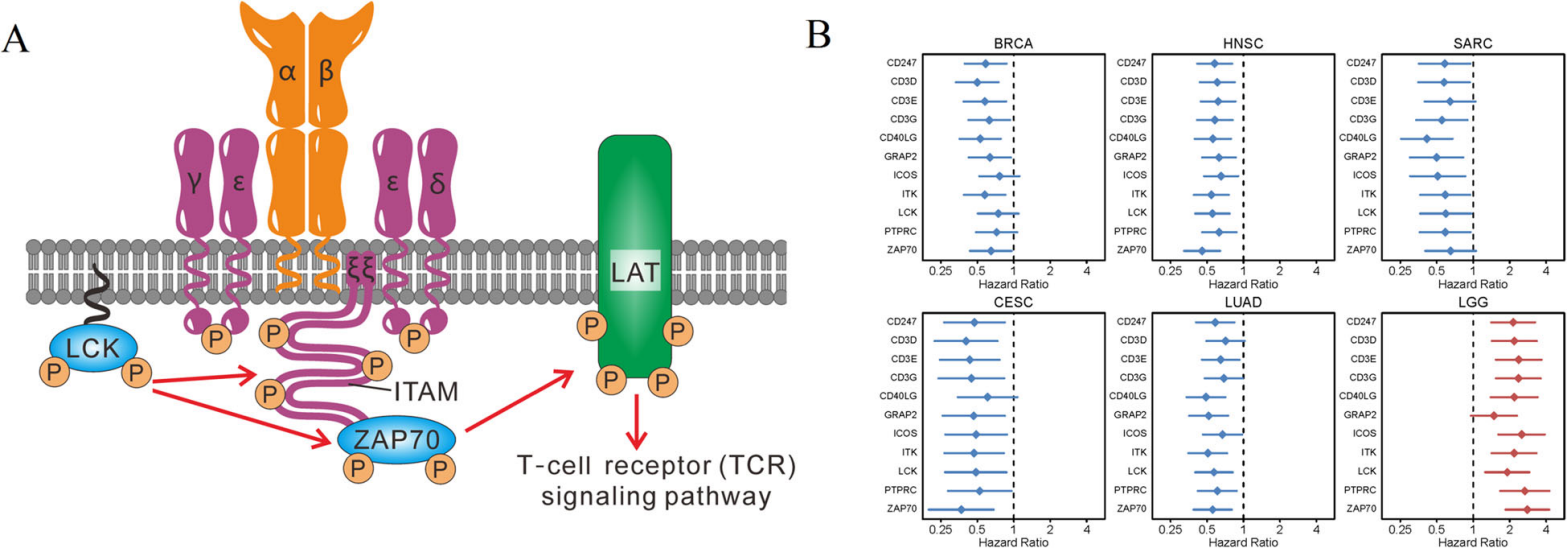
The TCR signaling pathway plays an opposite role in the OS of 6 cancer types. **a** TCR signal-triggering module. TCR signaling activation is initiated by the recognition of cognate peptide–MHC molecules, and then LCK phosphorylates the ITAMs (including the CD3γ chain (CD3G), the CD3δ chain (CD3D), the CD3ε chains (CD3E), and the ζ-chains (CD247)). Activated ITAMs provide docking sites for the SH2 domains of ZAP-70, which is phosphorylated by Lck, allowing propagation of downstream signaling events. **b** HR of 11 PIGs involved in the TCR signaling pathway in 6 cancer types. All HRs were > 1 (risk factor) in LGG but<1 (protective factor) in 5 cancer types (BRCA, CESC, HNSC, LUAD, and SARC)

The STRING database was used to explore interactions of the proteins encoded by the 48 mutual PIGs identified in the 6 cancer types. The STRING database confirmed a substantial potential interaction network, with a predominance of proteins involved in the TCR signaling pathway, primary immunodeficiency, Th17 cell differentiation, cytokine-cytokine receptor interaction, and Th1 and Th2 cell differentiation KEGG pathways (Fig. 2a). Eleven genes (ZAP70, PTPRC, LCK, ICOS, CD3E, CD3G, CD3D, ITK, CD247, CD40LG, and GRAP2) were identified as participating in the TCR signaling pathway (Fig. 2b). Among these genes, CD3E, CD3G, CD3D, and CD247 were immunoreceptors with tyrosine-based activation motifs (ITAMs). Following recognition of cognate peptide-MHC molecules, ITAMs are phosphorylated and activated by the SRC kinase family member LCK. Then, zeta-chain-associated protein kinase (ZAP70) is recruited to the activated ITAMs and phosphorylated by LCK, activating a signal transduction cascade that ultimately leads to T cell activation [33]. ZAP70, CD3E, CD3G, CD3D, and CD247 were classified into a ‘TCR signal triggering module’ by Acuto et al., which was crucial to the successful initiation of T cell activation [34]. The details are presented in Fig. 3a.

In this study, first, the HRs of the 11 genes participating in the TCR signaling pathway of 5 cancer types (BRCA, CESC, HNSC, LUAD, and SARC) were all < 1, but the HRs of the 11 genes in LGG were all > 1 (Fig. 3b). Among the 11 genes, all were protective PIGs (HR < 1 and FDR < 0.05) in HNSC, 10 were protective PIGs (HR < 1 and FDR < 0.05) in CESC and LUAD, 9 were protective PIGs (HR < 1 and FDR < 0.05) in SARC, 8 were protective PIGs (HR < 1 and FDR < 0.05) in BRCA, and 10 were risk PIGs (HR > 1 and FDR < 0.05) in LGG. Second, 6 (LCK, ZAP70, CD3E, CD3G, CD3D, and CD247) out of 11 PIGs played crucial roles in activating T cell activation. Third, two drugs (pembrolizumab and nivolumab) against PD1 have been used for the treatment of patients with metastatic non-small-cell lung cancer [35, 36], recurrent or metastatic HNSC [37, 38], and recurrent or metastatic cervical cancer [39]. Therefore, TCR signaling pathway was associated with improved OS in the 5 cancer types (BRCA, CESC, HNSC, LUAD, and SARC) but with decreased OS in LGG. The Kaplan-Meier survival curves for the 11 genes (ZAP70, PTPRC, LCK, ICOS, CD3E, CD3G, CD3D, ITK, CD247, CD40LG, and GRAP2) in the 6 cancer types are presented in Additional file 1: Figure S1.

## Discussion

Investigating the efficacy of novel anticancer strategies based on immunotherapeutics requires a comprehensive understanding of the association between immune genes and OS. In this paper, we studied the relationship between OS and immune genes in a collection of nearly 8000 patients across 22 cancer types. Prognostic genes and PIGs of each tumor type were obtained. Functional enrichment analysis was then used to identify the relevant KEGG pathways of prognostic genes. The results demonstrated that both the number of PIGs and the HR of PIGs varied greatly with tumor type. In addition, 6 of 22 cancer types (BRCA, CESC, HNSC, LUAD, SARC, and LGG) were significantly enriched in multiple (≥5) immune system-related pathways. Among these 6 cancer types, 48 common PIGs were identified in at least 5 cancer types. Eleven PIGs were confirmed to participate in the TCR signaling pathway according to the STRING database. High-level expression of the PIGs participating in the TCR signaling pathway was associated with improved OS in 5 cancer types (BRCA, CESC, HNSC, LUAD, and SARC), but with decreased OS in LGG. Overall, immune genes played a diverse role in OS across different cancer types.

Previous studies have shown that the immune system plays paradoxical roles during cancer development [5]. Balkwill et al. and Coca et al. found that extensive infiltration of NK cells correlated with a favorable prognosis in patients with gastric [40] or colorectal carcinoma [41]. On the other hand, the study of Leek et al. found that macrophage infiltration was associated with poor prognosis in human breast carcinoma [42]. Moreover, multiple lines of evidence suggest that individuals who are prone to chronic inflammatory diseases have an increased risk of cancer development [43]. In this study, we found that the number of PIGs and the HR of PIGs varied greatly with tumor type. For example, protective PIGs (HR < 1) accounted for a large proportion of prognostic genes in 5 cancer types (BRCA, CESC, HNSC, LUAD, and SARC). In contrast, risk PIGs (HR > 1) accounted for a large proportion of prognostic genes among 9 cancer types (COAD, GBM, KIRC, KIRP, LGG, LUSC, OV, PAAD, and STAD). In addition, there was no significant difference between the number of protective PIGs (HR < 1) and risk PIGs (HR > 1) in BLCA and LIHC, and the ratios of PIGs to prognostic genes were low. This finding suggested that activation of most immune genes was beneficial for the prognosis of patients in 5 cancer types (BRCA, CESC, HNSC, LUAD, and SARC) but detrimental to the prognosis of patients in 9 cancer types (COAD, GBM, KIRC, KIRP, LGG, LUSC, OV, PAAD, and STAD). The correlation between OS and immune genes in BLCA and LIHC was not obvious.

Immune disorders contribute to the tumor growth, and it has been known for over a century that T cells perform a major function in manipulating endogenous antitumor immunity [44, 45]. To date, six immune checkpoint antibodies have been approved by the FDA for the treatment of patients with melanoma, lung cancer, bladder cancer, stomach cancer, renal cell cancer, head and neck cancer, and Hodgkin’s lymphoma [17–22, 46]. However, the potential therapeutic value of immune checkpoint inhibitors in other cancer types has yet to be confirmed in clinical trials. The results of this study demonstrated that the prognostic genes of 6 cancer types (BRCA, CESC, HNSC, LUAD, SARC, and LGG) correlated significantly with the immune system. However, the PIGs in these 6 cancer types played a distinct role in OS. Most PIGs were protective factors in the prognosis of 5 cancer types (BRCA, CESC, HNSC, LUAD, and SARC) but were risk factors in LGG. Eleven PIGs (ZAP70, PTPRC, LCK, ICOS, CD3E, CD3G, CD3D, ITK, CD247, CD40LG, and GRAP2) were mainly shared by the 6 cancer types (BRCA, CESC, HNSC, LUAD, SARC, and LGG) involved in the TCR signaling pathway. Six genes (ZAP70, LCK, CD3E, CD3G, CD3D, and CD247) played a key role in triggering the TCR signaling pathway [34]. The results of this study indicated that high expression levels of the PIGs related to the TCR signaling pathway were associated with poor OS in LGG but long-term OS in 5 cancer types (BRCA, CESC, HNSC, LUAD, and SARC). Our findings are consistent with previous clinical observations that two drugs (pembrolizumab and nivolumab) against PD1 improved OS in patients with metastatic non-small-cell lung cancer [35, 36], recurrent or metastatic HNSC [37, 38], and recurrent or metastatic cervical cancer [39]. These results suggested that TCR signaling pathway was associated with improved OS in 5 cancer types (BRCA, CESC, HNSC, LUAD, and SARC) but with decreased OS in LGG. Therefore, an opposite effect of the TCR signaling pathway on the OS of different cancer types should be seriously considered in immunotherapy.

Cytotoxic T lymphocyte-associated antigen 4 (CTLA4) and programmed cell death protein 1 (PD1) are two immune-checkpoint receptors that have been clinically targeted for cancer immunotherapy [16]. Both CTLA4 and PD1 are inhibitory receptors that negatively regulate T cell activation through distinct mechanisms [47]. In this study, there was no significant difference between the OS in patients with high expression of CTLA4 and PD1 and that of patients with low expression of CTLA4 and PD1 in most cancer types. Nevertheless, 11 PIGs related to the TCR signaling pathway were associated with opposite prognoses in 6 cancer types. High expression of 11 PIGs was associated with good prognosis in BRCA, CESC, HNSC, LUAD, and SARC but poor prognosis in LGG. Therefore, CTLA4 and PD1 can be used as targets of immunotherapy for the 5 cancer types but might not be appropriate for LGG. TCR signaling pathway activation is dependent on the kinase activity of SFKs, particularly LCK. There are three forms of LCK in T cells: a form with phosphorylation on only Tyr505 (inactive), a form with phosphorylation on only Tyr394 (active) or a form with phosphorylation on both Tyr394 and Tyr505 (active). LCK is positively or negatively regulated by a combination of autophosphorylation, the C-terminal SRC kinase CSK and the phosphatases CD45, protein tyrosine phosphatase nonreceptor type 22 (PTPN22) and PTPN6. Among them, CD45 can modulate LCK activation or inactivation by the dephosphorylation of Tyr505 of LCK or the dephosphorylation of Tyr394 of LCK [33, 48]. Therefore, CD45 can be deemed a gatekeeper of T cell activation. The pivotal role CD45 in dynamically regulating the activation of LCK makes it an attractive target for immunotherapy.

To investigate the differentially expressed PIGs in tumors compared to normal tissues among 22 cancer types, 14 cancer types with >10 control samples were selected for analysis. By intersecting the DEGs and PIGs, differentially expressed PIGs and their proportion in total PIGs were obtained (Additional file 4: Table S3). KIRC had the highest number of differentially expressed PIGs, 550. UCEC had the lowest number of differentially expressed PIGs, only 1. Since there were no PIGs (corrected *p*-value< 0.05) in 3 cancer types (ESCA, PRAD, and THCA), there were also no differentially expressed PIGs. The largest percentage of differentially expressed PIGs in all PIGs was LUSC and LIHC (79 and 63%, respectively). The smallest proportion was HNSC (39%). Among the other 7 cancer types (BLCA, BRCA, COAD, KIRC, KIRP, LUAD, and STAD), the proportion of differentially expressed PIGs was approximately half (50%~ 59%). In addition, the number of upregulated PIGs and downregulated PIGs among different cancer types was also compared. Eight cancer types (BLCA, BRCA, COAD, KIRC, KIRP, LIHC, LUAD, and STAD) had more downregulated PIGs than upregulated PIGs. In contrast, the numbers of upregulated PIGs in KIRC and HNSC were larger than the numbers of downregulated PIGs. In addition, in KIRC, the number of upregulated PIGs was twice that of downregulated PIGs. Since there was a small number of differentially expressed PIGs among the 14 cancer types, it was difficult to obtain a PIG signature among these cancer types.

It should be noted that this study utilized the genomic data rather than protein data to explore the relationship between OS and immune genes. Since genomics represents merely the first step towards an understanding of cellular and even higher-order functions, it is necessary to complement these results with a systematic analysis of the proteins. In addition, the heterogeneity of the cohort in terms of tumor stage or histology might contribute to the different prognoses across different tumor types. Prospective studies of homogenous cohorts will verify our findings.

## Conclusions

In summary, our integrated analysis provides a powerful avenue to comprehensively dissect the relationship between immune genes and OS. Furthermore, we found that TCR signaling pathways played a distinct role in OS in 6 cancer types (BRCA, CESC, HNSC, LUAD, SARC, and LGG). These findings will contribute to the improvement of cancer immunotherapy.

## Supplementary information

**Additional file 1: Figure S1.** Kaplan-Meier survival curves for the 11 PIGs (ZAP70, PTPRC, LCK, ICOS, CD3E, CD3G, CD3D, ITK, CD247, CD40LG, and GRAP2) involved in the TCR signaling pathway across 6 cancer types (BRCA, CESC, HNSC, LUAD, SARC, and LGG) with FDR (adjusted *p*-value) < 0.05.

**Additional file 2: Table S1.** Gene set enrichment results of prognostic genes in 22 cancer types.

**Additional file 3: Table S2.** HRs of 48 PIGs shared by 6 cancer types (FDR < 0.05).

**Additional file 4: Table S3.** Differentially expressed PIGs of 14 cancer types (FDR < 0.05).

## Data Availability

The datasets of human gene expression and clinical data of 22 cancer types supporting the conclusions of this article are available in the Broad Institute’s Genome Data Analysis Center (GDAC) repository (http://gdac.broadinstitute.org/) and the cBioPortal for Cancer Genomics repository (http://www.cbioportal.org/). The immune gene list supporting the conclusions of this article is available in ImmPort repository (https://immport.niaid.nih.gov) and InnateDB repository (http://www.innatedb.ca/).

## Abbreviations

BLCA: Bladder urothelial carcinoma
BRCA: Breast invasive carcinoma
CESC: Cervical squamous cell carcinoma and endocervical adenocarcinoma
COAD: Colon adenocarcinoma
DEGs: Differentially expressed genes
ESCA: Esophageal carcinoma
FDR: False discovery rate
GBM: Glioblastoma multiforme
HNSC: Head and neck squamous cell carcinoma
HR: Hazard ratio
ImmPort: The immunology database and analysis portal system
ITAMs: Immunoreceptors with tyrosine-based activation motif
KIRC: Kidney renal clear cell carcinoma
KIRP: Kidney renal papillary cell carcinoma
LGG: Brain lower grade glioma
LIHC: Liver hepatocellular carcinoma
LUAD: Lung adenocarcinoma
OS: Overall survival
OV: Ovarian serous cystadenocarcinoma
PAAD: Pancreatic adenocarcinoma
PIGs: Prognostic immune genes
PRAD: Prostate adenocarcinoma
SARC: Sarcoma
SKCM: Skin cutaneous melanoma
STAD: Stomach adenocarcinoma
TCGA: The Cancer Genome Atlas
TCR: T cell receptor
THCA: Thyroid carcinoma
THYM: Thymoma
UCEC: Uterine corpus endometrial carcinoma
