# Supplementary figures and images for: A systematic analysis of immune genes and overall survival in cancer patients
Source: BMC Cancer. 2019 Dec 16;19:1225. doi: 10.1186/s12885-019-6414-6 (PMC6915928; doi:10.1186/s12885-019-6414-6)

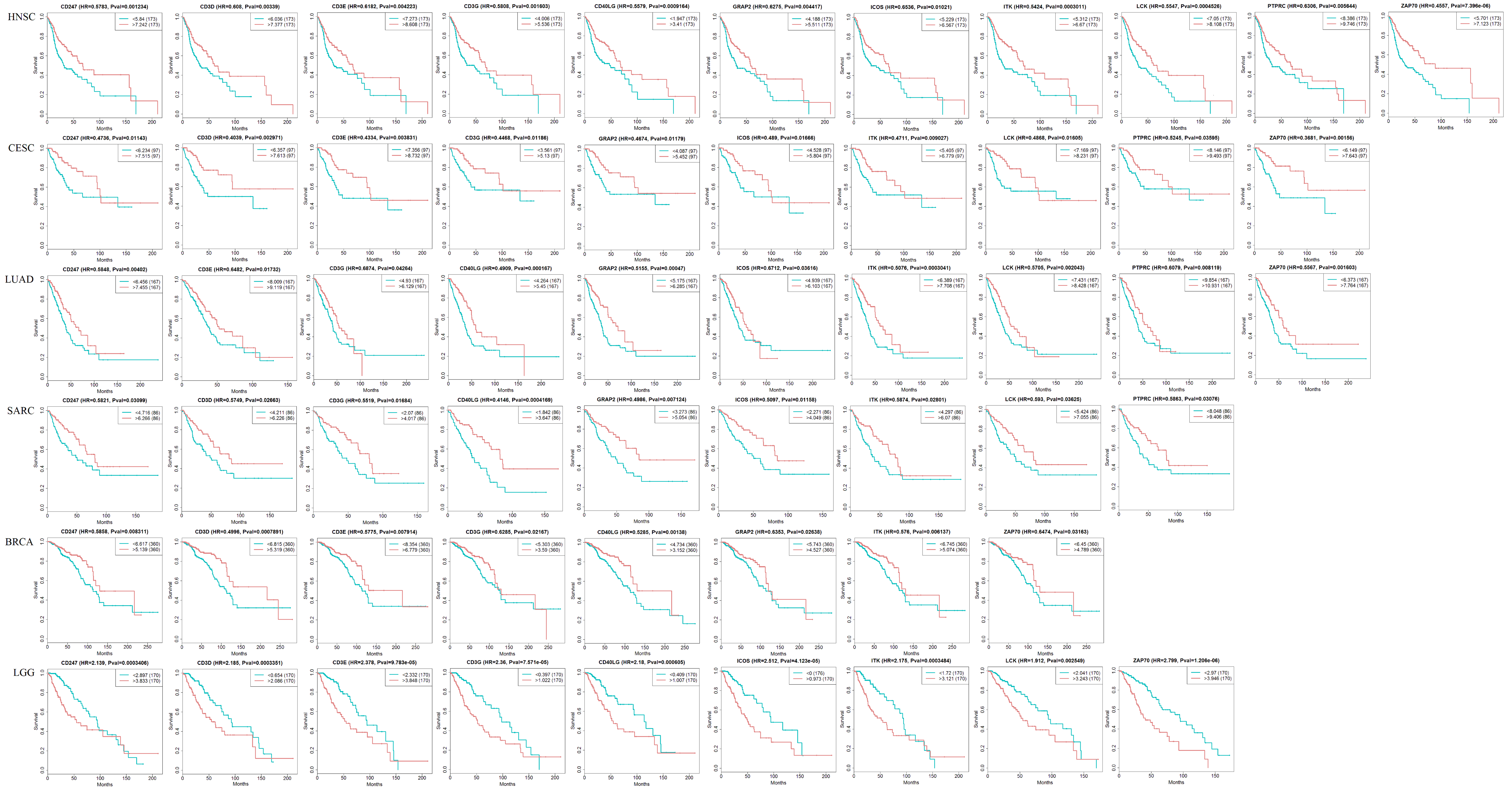

Supplement: Supplementary file 1 — Additional file 1: Figure S1. Kaplan-Meier survival curves for the 11 PIGs (ZAP70, PTPRC, LCK, ICOS, CD3E, CD3G, CD3D, ITK, CD247, CD40LG, and GRAP2) involved in the TCR signaling pathway across 6 cancer types (BRCA, CESC, HNSC, LUAD, SARC, and LGG) with FDR (adjusted p-value) < 0.05. [file 12885_2019_6414_MOESM1_ESM.tiff]
